# Children with autistic spectrum disorder can imagine actions— what can this reveal about the Broken Mirror Hypothesis?

**DOI:** 10.3389/fneur.2025.1490445

**Published:** 2025-01-23

**Authors:** Jessica Galli, Laura Dusi, Gioacchino Garofalo, Alessandra Brizzi, Michela Gritti, Federica Polo, Elisa Fazzi, Giovanni Buccino

**Affiliations:** ^1^Department of Clinical and Experimental Sciences, University of Brescia, Brescia, Italy; ^2^Unit of Child Neurology and Psychiatry, ASST Spedali Civili of Brescia, Brescia, Italy; ^3^Department of Philosophy, University of Bologna, Bologna, Italy; ^4^Faculty of Medicine and Surgery, University “Vita-Salute” San Raffaele, Milan, Italy; ^5^IRCCS San Raffaele, Milan, Italy

**Keywords:** autism spectrum disorder, motor imagery, mirror neuron system, Broken Mirror Hypothesis, motor system

## Abstract

**Objective:**

This study investigated whether children with Autistic Spectrum Disorder (ASD) can imagine object directed actions similarly to their typically developed (TD) peers.

**Study design:**

We tested the ability to imagine goal directed actions in children with ASD (*n* = 18) and TD (*n* = 18) peers by means of VMIQ-2 questionnaire and a novel behavioral task, in which children were requested to imagine some daily actions, after seeing them through videoclips presented on a computer screen. Observed actions lasted 4 s and children were requested to follow the same time course during imagination. During this motor imagery (MI) task, children were interrupted at a specific timepoint (e.g., at 1.5 s) from the beginning of the task. Afterwards, they had to select one of two frames extracted from the videoclips: one showed the correct timepoint at which the imagined action was stopped, the other depicted an earlier or later timepoint. Children had to press the key associated to the correct frame to provide their responses.

**Results:**

Both groups performed similarly in the questionnaire and in the novel MI task, where they showed the same error rate. Errors distribution suggests that all children exploited a similar strategy to solve the task, being errors mainly distributed in judging the later frame.

**Conclusion:**

These findings support the view that children with ASD can imagine actions similarly to their TD peers. These results do not fully support the Broken Mirror Hypothesis and may encourage the use of MI as a cognitive strategy in the rehabilitation of autism.

## Introduction

Autism spectrum disorder (ASD) is a life-long neurodevelopmental condition characterized by impairment in social communication and interactions, co-occurring with restricted interests and repetitive stereotyped behaviors (1). Furthermore, deficits in action observation and recognition, imitation, praxis and in the ability to understand others’ action intentions have been reported (2–5). One of the most influential, although still debated, theoretical framework to explain impairment in action processing and understanding, social interactions and communication found in children with ASD, maintains that a developmental disorder of the mirror neuron system (MNS) may play a causal role in ASD. In humans, the MNS consists of strictly connected frontal and parietal areas that are active during the execution of goal directed actions, during the observation and recognition of actions in the same category as well as during internal re-enactment of those actions like in motor imagery (6–9). More recent papers [for review see (10)] have also shown that the fronto-pariental areas that build up the MNS are also recruited during the processing of sentences expressing an action content. Hence, a developmental dysfunction of MNS has been proposed to explain the core aspects of ASD, especially as far as the social and communicative deficits. This explanation is often referred to as the Broken Mirror Hypothesis (BMH) (11).

The BMH has been especially investigated in imitation behavior and action observation (12). Less attention has been paid to motor imagery (MI). However, if one admits that the dysfunction of the MNS is at the root of the ASD symptoms (11), we can speculate that also other motor related cognitive functions, like MI, should be affected. In details, MI describes the ability of human beings to mentally rehearse simple or complex actions that are not accompanied by overt body movements (13). It implies the effort of individuals to imagine themselves while performing a given action. Empirical evidence supports the view that MI shares the same neural basis and mechanisms involved during execution, observation and verbal description of imagined action [for review see (8, 10, 13)]. The time course of the imagined action overlaps that of the executed action, at least in adults (14, 15).

In the present study we assessed the ability of ASD children to imagine goal directed actions. The aim of the study was twofold: first, we were interested in assessing MI ability in ASD children to provide further insights on the validity of the BMH in explaining ASD deficits; second, to assess whether MI may be exploited as a rehabilitation tool in ASD deficits, as it is in other childhood neurological disorders.

## Methods

### Study design and ethics

This was a randomized case–control study. We tested the ability to imagine goal directed actions related to daily activities in two matched groups of children: ASD and healthy Typical Developed (TD), respectively. We did so by means of a well-established MI questionnaire [Vividness of Movement Imagery Questionnaire-2 – VMIQ-2 (16)], and a novel, ecological task (MI task, see below for a detailed description), where we asked children to imagine goal directed actions following exactly their time course prompted by the observation of the same actions on videoclips. In the MI task children were asked to observe a goal directed action. Their capacity to imagine themselves performing the seen movement (asynchronous motor imagery) was subsequently assessed, focusing not only on the goal, but also on the temporal aspect (i.e., the time course) and duration of the action itself. Note that while the VMIQ-2 provided us with a subjective description of children’s capacity to imagine actions, the novel MI task provided us with a more objective index of the actual MI capacity by participants.

The study was approved by the Review Board of ASST Spedali Civili of Brescia (Comitato Etico di Brescia, ID number: NP 5749).

### Participants

All children referred to the Unit of Child and Adolescent Neurology and Psychiatry at ASST Spedali Civili (Civil Hospital) of Brescia with a diagnosis of ASD from December 2022 to March 2023 were eligible. Inclusion criteria were: (1) Diagnosis of ASD, level 1, in accordance with the Diagnostic and Statistical Manual of Mental Disorders, 5th Edition (DSM-5); (2) age between 6 to 15 years; (3) Full Intelligence Quotient >70 standard score (s.s.). Exclusion criteria were the presence of major visual and/or auditory deficits and drug treatment acting on the central nervous system. A total of 18 children (mean age 11.3 years, SD 2.9; 12 males, 6 females) met the inclusion/exclusion criteria and were enrolled. Full details of all enrolled children are shown in Table 1. Eighteen healthy children, matched by age, sex and school level, were also recruited as a control group (mean age 11.2 years, SD 3.0; 12 males, 6 females). Before entering the study, the parents of each child gave written informed consent.

**Table 1:** Demographic data and clinical features of participants.

Patient no.	Sex (M/F)	Age (years, months)	ADOS-II comparative score	CASD score	WISC-IV (FIQ)	VABS-II (total score)
1	M	8,11	6	17	98	74
2	F	7,10	6	17	99	77
3	M	10,2	4	14	108	82
4	M	14,6	7	13	101	38
5	M	14,2	6	16	72	81
6	M	6,6	5	12	71	62
7	M	13,9	7	14	84	34
8	M	10,11	5	15	106	66
9	M	15,6	7	19	97	66
10	F	7,4	10	10	108	76
11	F	12,4	4	14	128	98
12	F	15,11	5	15	93	67
13	M	10,8	6	12	110	74
14	M	10,3	7	14	100	72
15	M	14,1	7	21	104	76
16	F	10,5	4	10	91	74
17	M	7,7	6	11	107	78
18	F	11,11	4	11	78	67

M, male; F, female; ADOS-II, Autism Diagnostic Observation Schedule, 2nd Edition; CASD, Checklist for Autism Spectrum Disorder; WISC-IV, Wechsler Intelligence Scale for Children; FIQ, Full Intelligence Quotient; VABS-II, Vineland Adaptive Behavior Scales-II.

The diagnosis of ASD was made in accordance with the DSM-5 and performed by a multidisciplinary team including a child neuropsychiatrist and an experienced child psychologist. Additionally, ASD symptoms were examined using the Autism Diagnostic Observation Schedule, 2nd Edition – ADOS-II (17) and the Checklist for Autism Spectrum Disorder – CASD (18).

The cognitive level was evaluated using the Wechsler Intelligence Scale for Children – WISC-IV (19), specifically we collected the following scores: Full Intelligence Quotient (FIQ), Verbal Comprehension Index (VCI); Perceptual Reasoning Index (PRI); Working Memory Index (WMI) and Processing Speed Index (PSI). Adaptive functioning was assessed using the Vineland Adaptive Behavior Scales-2nd edition [VABS-II (20)].

### Clinical features

All participants had a diagnosis of ASD, level 1. According to ADOS-II, 17 participants had low/medium level of autistic symptoms (comparative score between 4–7) and 1 a high level (comparative score = 10); the mean of ADOS-II comparative score was 5.8 ± 1.5 (range 4–10). At CASD, 2 out of 18 children were in the non-autistic range, 9 in autism spectrum range and 7 in autistic range. The cognitive evaluation revealed that 4 participants had a borderline FIQ while the remaining 14 presented a normal level. The mean of FIQ was 97.5 ± 14.3 s.s. (range: 71–128 s.s.); mean VCI: 104.9 ± 16.7 s.s. (range 82–142), mean PRI 101.9 ± 15.2 s.s. (range 71–124); mean WMI 92.6 ± 16.3 s.s. (range 64–124), mean PSI 86 ± 12.6 s.s. (range 68–106). Adaptive functioning was impaired in 7 cases, borderline in 10 and normal in 1 case; the mean of total score was 70.1 ± 14.7 s.s (range 34–98). As regard VABS-II domains we observed that 12 participants had low scores (impaired: 6 cases; borderline: 6 cases) in communication (mean 76.2 ± 16.8, range 42–101 s.s.); 13 (impaired: 3 cases; borderline: 10 cases) in daily living skills (mean 79.3 ± 12.9, range 55–109 s.s.) and 16 (impaired: 7 cases; borderline: 9 cases) in socialization, (mean 68.1 ± 17.3, range 20–90 s.s.).

### Apparatus, stimuli, and procedure

The experiment was conducted in a dimly lit room at the U.O. of Childhood and Adolescent Neuropsychiatry of Brescia. The child participated in the experiment while seated in front of a computer screen that displayed the instructions and stimuli. Stimuli were four-second video clips of an actor carrying out typical daily tasks using various objects. We chose common activities that children are already familiar with.

Children were required to respond by pressing one of two keyboard keys (“Q” or “Page Up”) placed with respect to the children’s body midline. The keys were colored in yellow and red, respectively.

**Figure 1:**
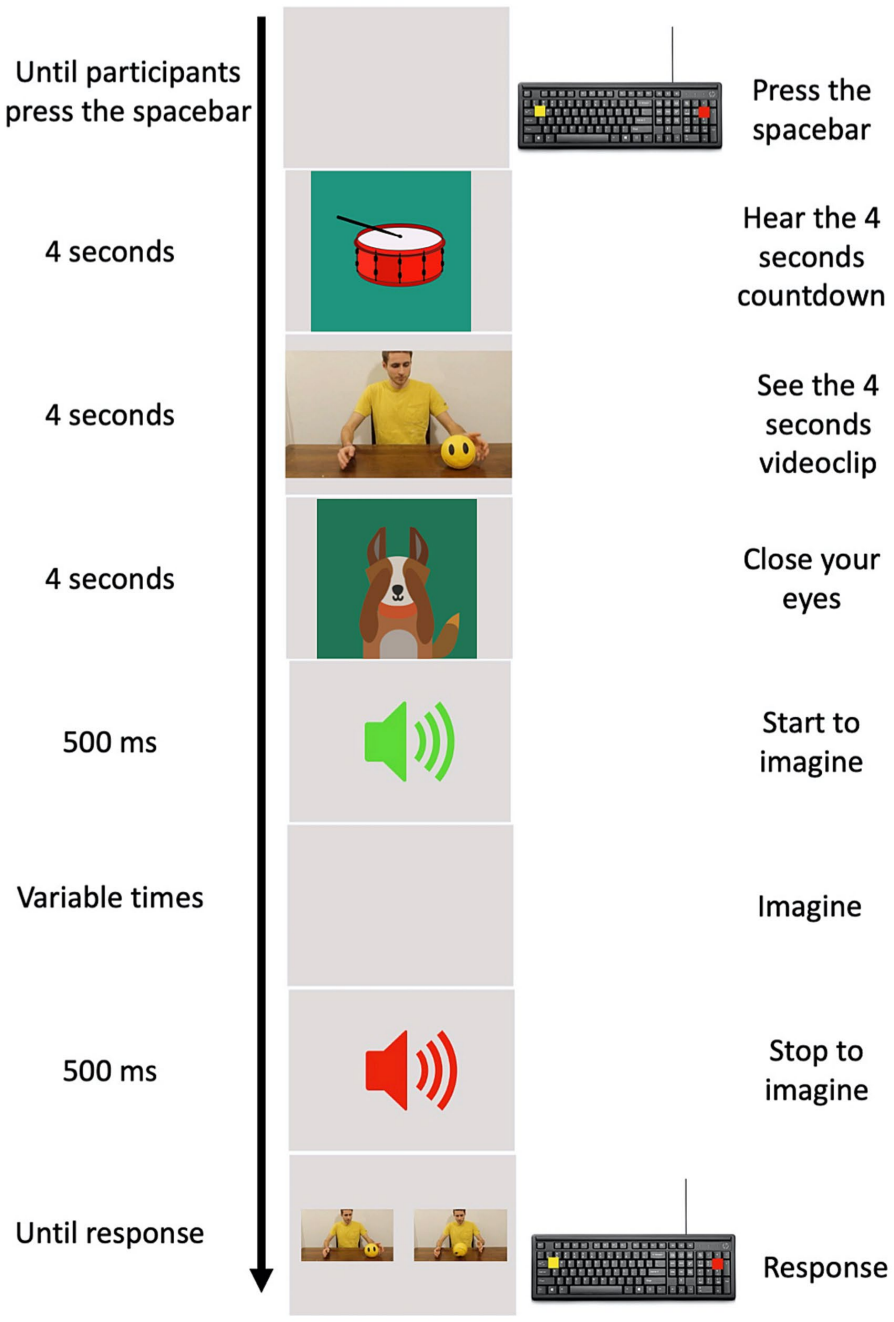
Experimental procedure. In the left column, the time of each event is reported. In the middle column, a pictorial example of the events is depicted. In the right column, the action requested by participants to provide their responses is reported.

The experimental task was implemented using PsychoPy 3.0. Ten practice trials were used to train the children. Practice trials were not further examined. An experimental trial started when the child pressed the spacebar. An animated tambourine that rhythmically beat 4 shots (1 at s) appeared on the screen to give an auditory and mental timing and prepare the child for the clip. A four second clip sequence appeared on the screen showing an actor, in a frontal position with respect to the observer, performing one of the chosen daily actions. The videoclip was replaced with a cartoon of a little dog, that placed its paws to its eyes and invited the child to close his/her eyes. An auditory signal (Start signal) instructed the children to begin imagining the previously seen action, in first person perspective, following the same time course. A second and different auditory signal (Stop signal) was provided at different randomized time intervals (1.5, 2, 2.5, 3, and 3.5 s) during motor imagery. The stop signal indicated the moment at which children had to stop to imagine the action and then re-open their eyes. Two images showing two frames of the previously seen action appeared on the monitor after the Stop signal. One of these frames coincided with the exact moment the action was stopped, the other depicted a moment 750 ms earlier or later than the exact one (Figure 1). The child was required to choose the frame representing the correct moment when the action was interrupted by pressing one of two colored buttons on the keyboard. We counterbalanced the position of the correct picture representing the exact moment in which the action was interrupted, presenting it on the screen either on the left or on the right. Children performed 80 trials (4 actions, × 5 different randomized time intervals at which the action could be interrupted, × 2 frames that could be either earlier or later than the exact one, × 2 positions of the correct frame). The task was designed to allow kids to take a break anytime they needed to avoid possible mental fatigue.

All children enrolled also completed the two scales of the VMIQ-2 designed to assess their self-reported capacity to imagine actions. In Scale 1 (EVI scale, External imagery) children are requested to imagine themselves performing an action from a third-person perspective; in Scale 3 (KIN scale, Kinesthetic imagery) they are requested to feel themselves performing an action.

### Analyses

The error rate was recorded and analyzed. We considered an error when the selected frame did not show the exact instant when the imagined action was stopped. R 4.2.0 was used to perform data analysis.

Given the design of the experiment and the characteristic of the errors distribution (binomial distribution), we modeled the data using a multilevel logistic regression. The selection of the model that best expresses the plausibility of our data with respect to the variables considered was made using the Bayesian index (BIC) (21). This allowed us to predict with equal probability the a-priori likelihood of the null hypothesis (H0) and of the alternative hypothesis (H1). Bayes weights are used to assess the model uncertainty, which can be considered analogous to an estimate of the probability that a given model is the best model that yields the data. As a result, if a model has a Bayes weight greater than 0.95, it is deemed the only valid data model. If no model meets this requirement, all models are sorted from best to worst, and the list is continued until the cumulative weight of Bayes exceeds 0.95, at which point the remaining models are dismissed. This defines a “confidence set” of 95% models, meaning we can be 95% sure that one of the models in the set is the best approximation to the data.

The full model has been implemented with Group (2 levels: ASD vs.TD) as a between-participant factor, and Time-point (2 levels: earlier vs. later than the correct frame) and Stop Time (i.e., 5 levels: 1.5, 2, 2.5, 3, and 3.5 s) as within-participant factors. Participants and videoclips were set as random effects. Model selection was performed using dredge function of MuMIn package.

VMIQ-2 scores have been analyzed running a between groups t-tests (TD vs. ASD) for both scales of the questionnaire.

## Results

**Figure 2:**
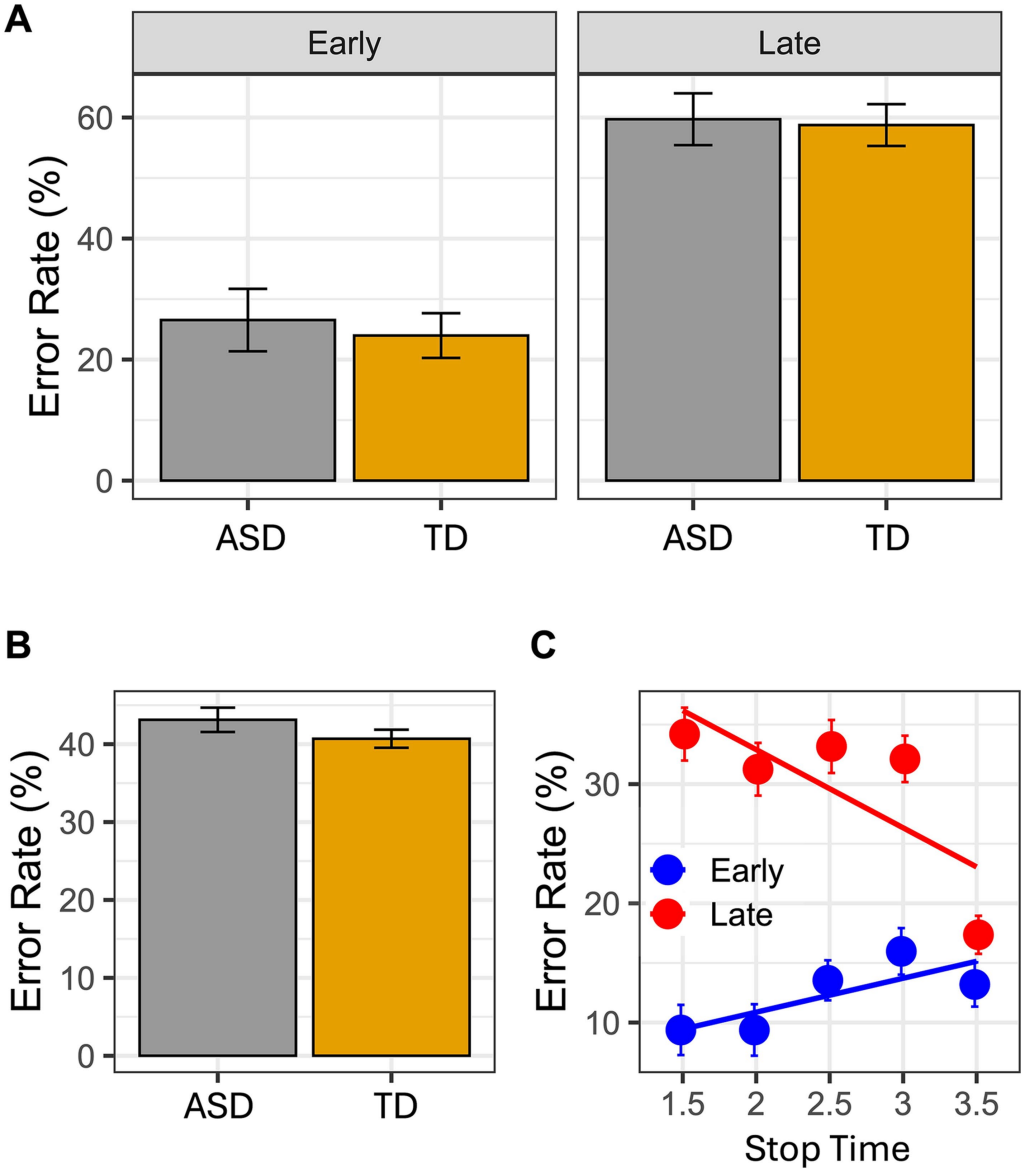
In the graphs it is reported the error rate index calculated as follows: incorrect response/(Correct response + Incorrect response). In **(A)** mean Error rate is reported as a function of Group and Time Point. In **(B)** mean Error rate is reported as a function of Group. Finally, the panel **(C)** reported the interaction between Time Point and Stop Time. Error bar referred to the standard error of means.

Bayesian analysis showed that the best model that yields our data is the one including the reliable effects Time-point and Stop Time, and the interaction between them (BIC = 3553.1, Bayes weight = 1). For the results of the best model see Tables 2, 3. Intriguingly, no between group difference occurred: both groups made the same errors as shown (Figure 2B). Moreover, this pattern emerged even when investigating the interaction between Group and Time-point (Figure 2A; Table 3), confirming the result of the model selection.

**Table 2:** Best model results.

	Estimate	Std. Error	z-value	Pr(>|z|)
Intercept	1.22644	0.13546	9.054	< 0.001
Time-point	−2.8299	0.20222	−13.994	< 0.001
Stop Time	−0.27896	0.03928	−7.101	< 0.001
Time-point: Stop Time	0.43422	0.05888	7.374	< 0.001

**Table 3:** Descriptive statistics.

	Before			After		
	Mean	SD	Std. Err.	Mean	SD	Std. Err.
ASD	26.5	21.9	5.2	59.7	18.2	4.3
Control	24.0	15.3	3.7	58.8	14.6	3.4

Results of the VMIQ-2 did not show any significant differences between groups [Scale 1—EVI: *t*(1, 27.551) = −0.48, *p* = 0. 63; Scale 3—KIN: *t*(1, 27.68) = 0.96, *p* = 0.36], confirming that both healthy children and children with ASD can image the actions described in the questionnaire in both scales (EVI: Mean ASD = 3.89, SEM = 0.24; Mean TD = 4.02, SEM = 0.14; KIN: Mean ASD = 3.83, SEM = 0.25; Mean TD = 4.11, SEM = 0.15).

## Discussion

The present findings show that children with ASD have the ability to imagine goal directed actions like their typically developed peers. The results of the VMIQ-2 (subscales EVI and KIN), as well as the results obtained in the MI task, did not show any significant difference between groups. Children with ASD and their peers made the same number of errors. In details, errors mostly occurred when children had to judge the picture presenting the later frame. These results overlap those found in a similar study, where children with cerebral palsy and their normally developed peers were included (22). It is worth stressing that at difference with Galli et al.’s study, in the present one the best model that yields our data include also the interaction between Stop Time and Time-point. As shown in Figure 2C, the general pattern of errors is further supported by this interaction. In other words, in both groups the timing of the imagined actions was faster than the timing of the seen actions. These findings strongly suggest that, while imagining actions, both groups were strictly anchored to the goal of the action (see the error rate in the 3.5 s Stop Time), so that both groups tended to anticipate the final part of it (i.e., hand-object interactions) and the imagined action resulted globally faster than the observed one.

One of the most intriguing explanations concerning the neurophysiological basis of the ASD relies in the dysfunction of MNS (23). Studies assessing the impairment of the MNSFare clic o toccare qui per immettere il testo., both in adults (24, 25) and children (2, 26, 27) with ASD, showed that patients presenting with this disorder are not able to understand observed actions on the basis of their motor features, and to interpret gaze and head movements of the agent. By means of transcranial magnetic stimulation (TMS) study, Theoret et al. (25) showed that in adults with ASD, as compared with healthy individuals during the observation of finger movements, there is no specific modulation of muscles involved in the execution of the same action, as revealed by induced motor-evoked potentials (MEPs). With the same technique, Cattaneo et al. (26) also showed that in children with ASD, as compared with healthy peers, there is no anticipatory activation of muscles involved in ingestive actions during the observation of bringing to the mouth actions. In an fMRI study, where ASD and TD adolescents were asked to encode goal and no-goal directed actions, results revealed that MNS was not active in ASD group, suggesting a global deficit in this system (28). Dapretto et al. (11), extended this impairment of the MNS also to the motor components of the facial expressions useful to recognize emotions. Taken together these findings support a hard view of the role of MNS in the social and affective dysfunctions found in children with ASD.

The BMH would also imply an impairment in other motor related cognitive functions, as for example MI. However, the present findings showed that in children with ASD, the MI ability seems to be preserved. Hence, these findings do not completely fit with the BMH. In a similar vein, Hamilton (29) showed that children with ASD are able to imitate actions. Furthermore, in a Magnetoencephalography (MEG) study (24) authors found that during the observation of lip movements, in ASD participants there was a typical recruitment of areas belonging to MNS, although delayed in comparison to healthy adults. Overall, these pieces of empirical evidence provide mixed results that do not fully support the BMH. More recently, Hamilton (29, 30) has forwarded a mild view of the involvement of MNS in ASD deficits. In this view, two integrated models have been proposed: Emulation and Planning-Mimicry model (EP-M) and Social Top-down Response Modulation model (STORM). The main tenet of the EP-M model is that, within the MNS, the only impaired component is the one mediating the processing and imitation of no-goal directed actions, which require the reproduction of observed action sequences with specific kinematic features. Conversely, the component allowing the processing and imitation of goal-directed actions, is spared (29). Complementary to this, rather than a dysfunction within the MNS itself, the STORM model proposes that ASD symptoms stem from developmental impairments in the cerebral areas (e.g., medial PreFrontal Cortex, mPFC) that modulate the MNS, providing a proper contextualization and the social meaning to actions.

Since in our experiment we asked participants to motorically imagine goal-directed actions, our data fully support the EP-M model, reinforcing further the notion that in children with ASD level 1, the motor representations of goal directed actions are functionally preserved. Accordingly, a recent fMRI study (31) showed an intact ability in mental simulation of goal directed actions as compared to no-goal directed actions. The latter seem more demanding for ASD individuals, as revealed by a stronger activation of MNS during their processing. The authors suggested that this stronger activation may be due to a greater difficulty for ASD participants in processing this kind of actions.

In contrast with the present findings, early studies assessing the MI in ASD population have shown an impairment in this cognitive function (3, 32), leading to a general claim that MI in this population is more affected than the ability to actually execute and coordinate actions.

In our view, the different results may be related to the tasks that participants had to solve. In earlier studies, MI was assessed by means of rotational, laterality judgment and circles-lines tasks. These tasks also assess other cognitive domains like visual imagery, manual coordination, spatial perspective taking, and the process of object-related features. Moreover, in general these tasks used response times to assess MI ability, while in our task we focused on the capacity of children to follow, during MI, the exact time course of the actions shown in the videoclips. This in the attempt to limit the influence of other cognitive processes potentially affecting the response times and error rate, as suggested by Souto et al. (33).

Beyond its contribution to the ongoing debate on the validity of the BMH to explain clinical features of ASD, in our view the results of the present study are relevant for its clinical implications in the rehabilitation of children with ASD. Motor imagery has been considered for years a valid tool in motor learning in different contexts and in neurorehabilitation, including the rehabilitation of motor impairment in several childhood neurological disorders [for very recent reviews see (34, 35)]. The potential use of MI as a cognitive strategy for the rehabilitation of specific deficits in autism is debated [see (36) for review] also because of the non-univocal empirical results on the capacity of children with ASD to imagine actions. The present findings, by showing that children with ASD level 1 are able to imagine actions in a manner similar to their healthy peers, support the view that MI can be exploited as a rehabilitative strategy for the recovery of motor functions in these children.

Despite the results of the present study seem to be relevant from a theoretical point of view because they may contribute to the ongoing debate on the validity of the BMH in explaining clinical features of ASD, as well as for its clinical implications, some limitations should be underlined. Our sample was rather small and indeed future, possibly multicentric studies should aim at enlarging the number of children recruited and assessing the reliability of the task we proposed. Moreover, as for other studies of similar kind, our inclusion/exclusion criteria were rather stringent, and this limits the generalization of the results across the heterogenous autism spectrum disorder. In fact, these results apply to ASD children level 1 and cannot be generalized to more severe spectrum of ASD. A further limitation may be found in the questionnaire (VMIQ-2) used to assess MI in a subjective manner. Even though we used this questionnaire in a previous study assessing the MI in children with Cerebral Palsy (22), it is not validated for a population at this age. However, to the best of our knowledge, there are no validated scales for children aged 7 to 15 like the ones we enrolled. Moreover, we did not assess the cognitive level in TD children; however, they attended the school successfully and never revealed some cognitive impairments in everyday life activities. Finally, in this study, we assessed the ability to imagine only goal-directed actions, an action category that seems to be preserved in line with EP-M (29, 30). Future studies should assess the capacity of children with ASD to imagine others type of actions like, for example, mimicked actions and those expressing social conventions.

In conclusion, there is increasing evidence for a milder view of the involvement of MNS in both adult and pediatric populations with ASD moving to reconsider the BMH (37, 38) and the role of MNS in the physiopathology of ASD. The evidence that MI seems to be a preserved function in children with ASD level 1 open the way and support the use of this cognitive strategy as a potential rehabilitation tool in this challenging neurodevelopmental condition.

## Data Availability

The raw data supporting the conclusions of this article will be made available by the authors, without undue reservation.
